# Replacement bisphenols, phthalates, and micro-/nanoplastic exposures in human reproductive endocrinology

**DOI:** 10.3389/fendo.2026.1901581

**Published:** 2026-09-18

**Authors:** Elif Ganime Aygün, Edis Kahraman, Seyma S. Celina, Engin Oral

**Affiliations:** 1 Department of Obstetrics and Gynecology, Acıbadem Mehmet Ali Aydınlar University, Atakent Hospital, İstanbul, Türkiye; 2 Center for Infectious Animal Diseases, Czech University of Life Sciences Prague, Prague, Czechia; 3 Department of Obstetrics and Gynecology, School of Medicine, Acıbadem Mehmet Ali Aydınlar University, İstanbul, Türkiye; 4 Department of Obstetrics and Gynecology, Norrland University Hospital, Umeå University, Umeå, Sweden

**Keywords:** endocrine-disrupting chemicals, microplastics, nanoplastics, phthalates, replacement bisphenols, reproductive endocrinology, steroidogenesis

## Abstract

Restrictions on bisphenol A (BPA), continued exposure to phthalates, and the emergence of micro- and nanoplastics (MNPs) have altered the plastic-associated exposure landscape relevant to human reproduction. Existing reviews commonly address these exposures separately or summarize broad reproductive outcomes. This review instead develops a comparative mechanistic framework centered on two connected mechanisms: disruption of gonadotropin-responsive steroidogenesis and oxidative stress, inflammation, and barrier dysfunction. Human biomonitoring, reproductive-tissue, fertility-clinic, and epidemiological evidence are prioritized, while experimental studies are used to resolve mechanisms that cannot be tested directly in humans. Replacement bisphenols can reproduce BPA-like estrogenic, anti-androgenic, and steroidogenic effects, but their actions are analogue-, tissue-, dose-, and time-dependent. Selected phthalates and metabolites have been shown in experimental and ex vivo studies to impair androgen synthesis through altered gonadotropin signaling, cholesterol transport, mitochondrial function, and steroidogenic enzymes. MNPs are heterogeneous particulate exposures whose biological effects depend on polymer composition, size, shape, surface properties, weathering, corona formation, additives, and co-contaminants. Experimental studies using specific MNP models suggest mainly indirect effects on steroidogenesis through particle uptake, altered gonadotropin-responsive signaling, organelle stress, inflammation, and barrier injury. Despite different initiating mechanisms, evidence across the three exposure classes implicates steroidogenesis, redox regulation, inflammatory signaling, and reproductive tissue barriers, although the strength and directness of evidence differ. Human evidence is strongest for phthalates, emerging and analogue-specific for replacement bisphenols, and still methodologically limited for MNP-related clinical outcomes. The framework separates direct catalytic inhibition from altered expression, tissue detection from toxicity, and mechanistic plausibility from demonstrated human effect, supporting targeted research, cautious clinical communication, safer chemical substitution, and MNP exposure mitigation.

## 1 Introduction

Human reproductive function depends on tightly coordinated hormonal signaling, gonadal steroidogenesis, gametogenesis, implantation, placentation, and tissue remodeling. These processes are sensitive to environmental chemicals that interfere with receptor activity, enzyme function, metabolic signaling, or developmental programming. Concerns are reinforced by evidence of changing reproductive indicators and by the widespread detection of endocrine-disrupting chemicals (EDCs) in human populations (1–3).

The contemporary exposure landscape differs from the historical focus on BPA alone. Regulatory restrictions and consumer demand have increased the use of structurally related bisphenols, including bisphenol S (BPS), bisphenol F (BPF), and bisphenol AF (BPAF). At the same time, phthalates remain common in plastics, medical devices, personal-care products, and other applications. MNPs add a particulate dimension to this chemical burden because they may be internalized, interact with tissue barriers, and carry plastic additives or adsorbed contaminants (4–8).

These exposure classes should not be treated as toxicologically interchangeable. Replacement bisphenols and phthalates are soluble chemicals whose initiating actions may include receptor binding, transcriptional regulation, and direct or indirect perturbation of steroidogenic proteins. MNPs are heterogeneous particles whose biological identity depends on polymer type, size, shape, surface properties, weathering, aggregation, and the molecules adsorbed from their surrounding environment. Similar reproductive phenotypes can therefore arise from different initiating events (9–12).

The novelty of the present review lies in comparing these initiating events within a shared mechanistic structure. This review focuses on two connected mechanisms. The first is gonadotropin-responsive steroidogenesis, from receptor–cAMP signaling and mitochondrial cholesterol transport to specific catalytic steps. The second is oxidative stress and inflammatory injury, including mitochondrial and endoplasmic reticulum stress, loss of antioxidant capacity, barrier dysfunction, and regulated cell death. Testicular, ovarian, and placental observations are used as tissue-level tests of these mechanisms. Throughout the review, direct catalytic inhibition, altered gene expression, altered protein abundance, upstream signaling failure, tissue detection, and clinical association are treated as distinct forms of evidence.

## 2 Exposure characteristics and mixture context

### 2.1 Replacement bisphenols in relation to BPA

Replacement bisphenols retain the two phenolic-ring architecture that underlies several BPA-like endocrine activities. Structural and receptor studies show that many analogues interact with estrogen receptors and, in some cases, antagonize androgen signaling. However, the analogues do not form a uniform potency series. Receptor subtype preference, transcriptional response, and active concentration vary according to substitution pattern and assay system (9, 10, 13, 14).

Direct comparison with BPA is therefore essential. In adult human testis explants, BPA and several analogues produced compound-, concentration-, and time-dependent changes in testosterone or insulin-like factor 3 secretion, while the direction and magnitude of response differed among BPF, BPS, BPE, BPB, and bisphenol A diglycidyl ether (BADGE) (15). BPA directly inhibited human testicular HSD3B and CYP17A1 in microsomal assays, whereas replacement bisphenols have shown heterogeneous effects on aromatase and other steroidogenic targets (16, 17). BPA and BPS also produced partially different transcriptional responses in granulosa-cell models despite both disturbing steroid hormone secretion (18). These findings do not support the assumption that all substitutes are safer than BPA, but neither do they justify a single claim that every analogue is more potent or hazardous.

Human biomonitoring has detected replacement bisphenols in urine, blood, placenta, and breast milk, confirming widespread internal exposure (19–22). Yet the human reproductive evidence remains substantially smaller and more heterogeneous than the BPA literature. The most defensible interpretation is that replacement bisphenols can reproduce important BPA-like endocrine effects while also displaying analogue-specific mechanisms that require individual evaluation.

### 2.2 Phthalates as persistent plastic-associated EDCs

Phthalates remain pervasive because they are used in flexible plastics, medical products, personal-care products, and other consumer or occupational settings. Exposure is commonly assessed through urinary metabolites, although short biological half-lives, within-person variability, and multiple sources complicate exposure classification (5, 6). Their reproductive relevance is strongest for anti-androgenic developmental effects and male reproductive endpoints, but ovarian and pregnancy findings have also been reported with greater heterogeneity (23–26).

The term phthalates covers compounds with different side-chain lengths, metabolism, receptor activity, and enzyme effects. Selected parent phthalates have inhibited HSD3B or HSD17B3 in testicular microsomal assays, whereas the active metabolite mono-(2-ethylhexyl) phthalate (MEHP) has suppressed steroidogenic gene expression and mitochondrial function in experimental models (27–30). Consequently, class-wide statements should be qualified by compound, metabolite, dose, model, and developmental window.

### 2.3 Micro- and nanoplastics as heterogeneous particulate exposures

MNPs are not a single toxicant. They include different polymers—such as polyethylene (PE), polypropylene (PP), polystyrene (PS), polyethylene terephthalate (PET), polyvinyl chloride (PVC), polyamide, polyurethane, and polymethylmethacrylate—present as spheres, fibres, films, fragments, or irregular weathered particles. Primary particles may be manufactured at small size, whereas secondary particles arise through abrasion, thermal stress, ultraviolet exposure, and fragmentation. Polymer density, crystallinity, surface oxidation, charge, hydrophobicity, and additive composition influence environmental transport and biological interaction.

Size and shape alter the number of particles delivered at a given mass, surface-area-to-mass ratio, sedimentation, membrane contact, and uptake. In human epithelial models, uptake and stress responses differed among particle sizes and between spherical and fibre/fragment-shaped PS particles (31). Comparisons of PE, PP, PET, PVC, and PS likewise demonstrate polymer- and size-dependent transport and cellular interaction (32, 33). Most reproductive experiments, however, still use pristine monodisperse PS spheres, which do not represent the complexity of human exposure.

The biological surface of a particle also changes after entry into biological fluids. Proteins, lipids, and metabolites form a biomolecular corona that can alter aggregation, cellular recognition, uptake, and intracellular trafficking. Corona characteristics depend on particle size, surface chemistry, and the surrounding biological medium (34). A particle in follicular fluid, seminal plasma, or maternal blood may therefore behave differently from the same pristine material in simple culture medium.

MNP-associated toxicity may reflect at least three components: the physical and surface-mediated effects of the particle; chemicals that leach from additives, residual monomers, pigments, stabilizers, or degradation products; and environmental contaminants adsorbed to the surface. These components can act together, making it difficult to attribute an observed effect to the polymer backbone alone (12, 35). Reproductive studies should therefore characterize polymer identity, particle-size distribution, shape, surface charge, ageing, aggregation, additive content, and particle-free leachates wherever feasible.

Plastic-associated particles have been reported in human blood, placenta, breast milk, semen, seminal fluid, testicular tissue, and follicular fluid (36–43). More recent fertility-clinic studies have linked PE or PVC particles in reproductive fluids with exploratory measures of fertilization, sperm quality, oocyte quality, or ovarian reserve (44–46). These observations increase concern but do not yet establish causality. Sample sizes remain small, analytical platforms capture different size ranges, and contamination control and polymer confirmation are not uniform. Human MNP evidence must therefore be separated into detection, association, and demonstrated biological effect.

### 2.4 Mixture exposure and internal dose

Humans encounter bisphenols, phthalates, particles, and other contaminants concurrently through ingestion, inhalation, dermal contact, medical exposure, and occupational settings (**Figure 1**). Mixture effects may be additive, antagonistic, or synergistic, and non-monotonic dose responses complicate extrapolation from high-dose experiments (11, 47–49). MNPs may modify mixture toxicology by carrying plastic-associated chemicals or environmental pollutants and by changing their local distribution at epithelial or placental interfaces (12). The presence of common downstream targets does not prove that mixture components act identically, but it supports assessment of cumulative disruption at shared functional nodes.

**Figure 1:**
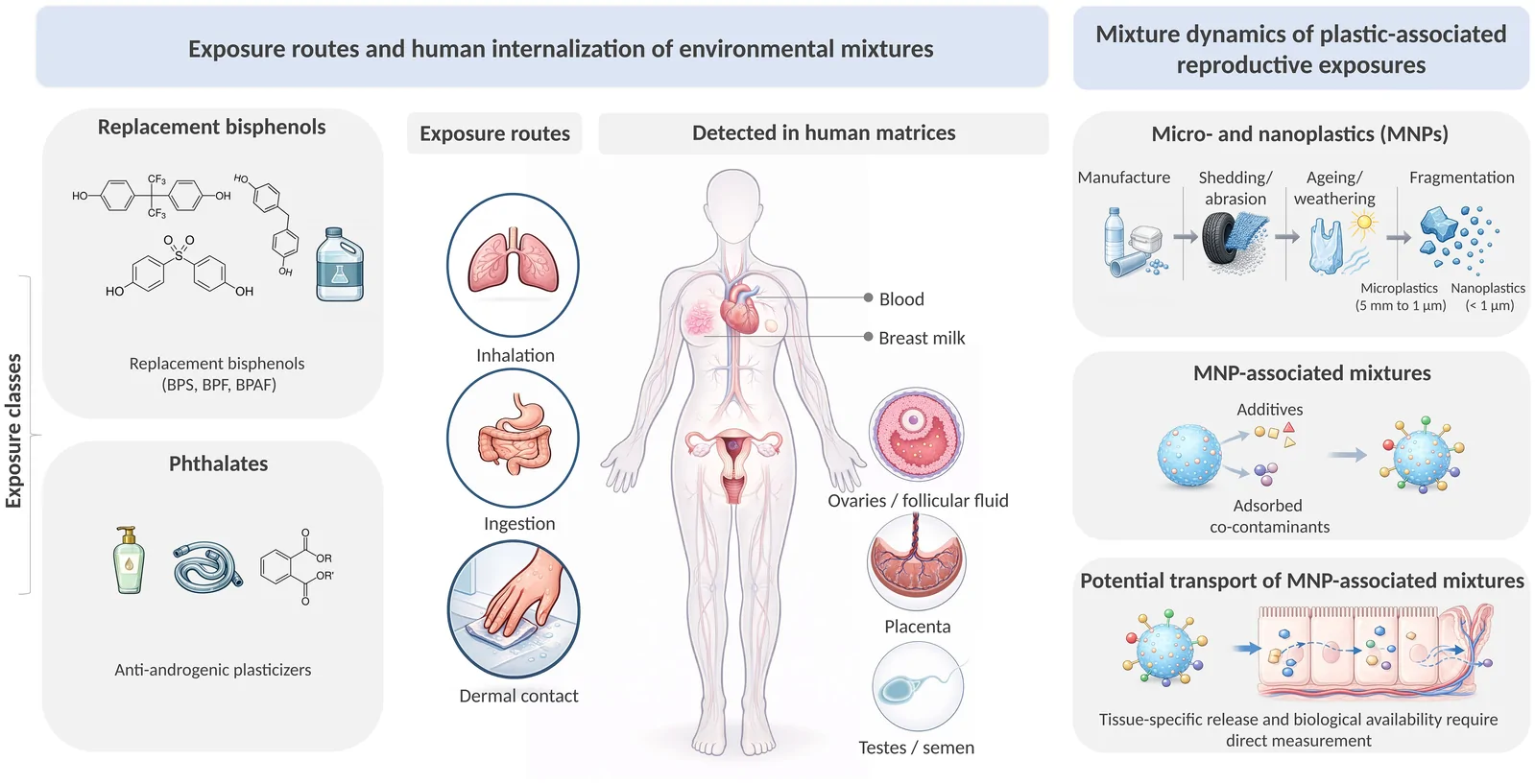
Exposure routes, internalization, and mixture dynamics of plastic-associated reproductive exposures. Replacement bisphenols and phthalates enter the body mainly through inhalation, ingestion, and dermal contact. Plastic-associated chemicals or particles have been reported in blood, breast milk, placenta, follicular fluid, semen or seminal fluid, and testicular tissue. Micro- and nanoplastics arise from manufacture, shedding, abrasion, ageing, weathering, and fragmentation and may carry additives and adsorbed contaminants across tissue barriers, although tissue-specific release and biological availability require direct measurement.

Combined exposures should therefore be interpreted at the level of specific pathways and endpoints rather than as a single toxicological entity. Components may converge on steroidogenesis, redox regulation, or barrier function while differing in potency, direction of effect, dose–response behavior, and exposure window. Mechanistic convergence does not necessarily indicate additive or synergistic toxicity, as interactions may be antagonistic or vary across endpoints. For mixtures containing MNPs, particle-mediated effects should additionally be distinguished from those of leached additives and adsorbed contaminants (11, 12, 47–49).

## 3 Disruption of gonadotropin signaling and steroidogenesis

In mixture settings, the pathways discussed below should be considered shared targets of potential cumulative disruption rather than evidence that all components act through the same initiating mechanism (**Figure 2**). Combined effects should therefore be interpreted according to compound, dose, tissue, timing, and endpoint.

**Figure 2:**
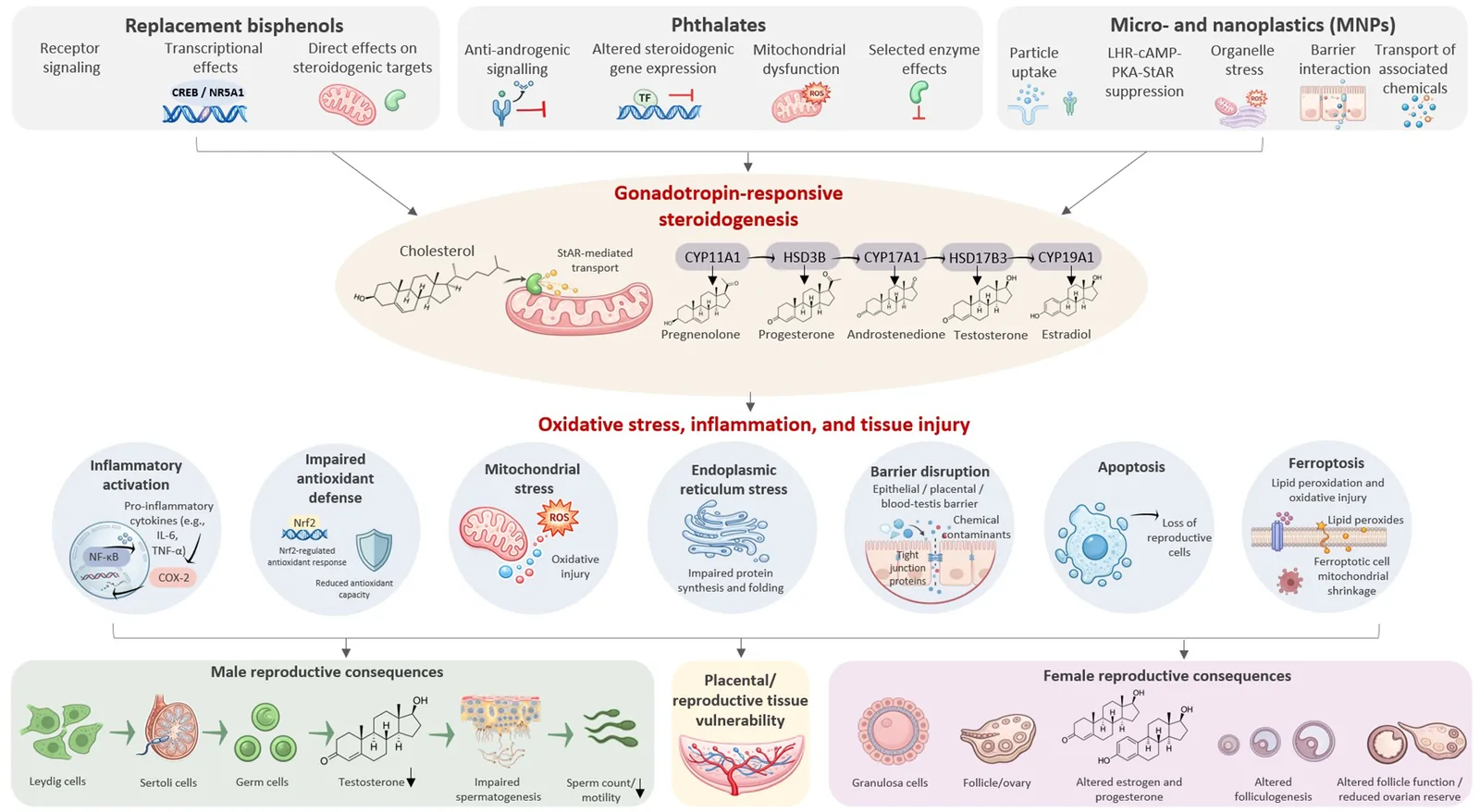
Comparative mechanistic framework for plastic-associated reproductive endocrine disruption. Replacement bisphenols, phthalates, and MNPs act through distinct initiating mechanisms that converge on gonadotropin-responsive steroidogenesis and oxidative stress, inflammation, and tissue injury. Key processes include altered receptor and transcriptional signaling, StAR-mediated cholesterol transport, effects on CYP11A1, HSD3B, CYP17A1, HSD17B3, and CYP19A1, mitochondrial and endoplasmic reticulum stress, impaired antioxidant defense, barrier dysfunction, apoptosis, and ferroptosis. These changes may impair male, female, and placental reproductive function. The steroidogenic pathway is simplified for clarity; intermediate metabolites and tissue-specific branches are not shown. In mixture settings, convergence on shared targets does not imply additive effects or toxicological equivalence.

### 3.1 Steroidogenesis as a shared mechanistic target

Gonadal steroidogenesis begins with gonadotropin-dependent signaling. In Leydig cells, luteinizing hormone (LH) activates LHCGR, adenylyl cyclase, cyclic adenosine monophosphate (cAMP), and protein kinase A. In ovarian theca and granulosa cells, luteinizing hormone and follicle-stimulating hormone regulate complementary steroidogenic functions through related cAMP-dependent pathways. CREB and NR5A1/steroidogenic factor-1 then regulate cholesterol transport and steroidogenic gene transcription (50, 51).

The steroidogenic acute regulatory protein (StAR) is a cholesterol-transfer protein. It facilitates movement of cholesterol to the inner mitochondrial membrane, where CYP11A1 converts cholesterol to pregnenolone with electron transfer through ferredoxin and ferredoxin reductase. HSD3B converts pregnenolone to progesterone and dehydroepiandrosterone to androstenedione. CYP17A1 provides 17α-hydroxylase and 17,20-lyase activities required for androgen synthesis. HSD17B3 converts androstenedione to testosterone in Leydig cells, whereas CYP19A1/aromatase converts androgen substrates to estrogens. HSD17B1 contributes to estradiol formation in ovarian and placental tissues (51).

Environmental disruption can occur at different levels: receptor binding; LHCGR/FSHR–cAMP–PKA signaling; StAR or steroidogenic-enzyme expression; protein synthesis, folding, or stability; mitochondrial substrate and electron transfer; or direct catalytic inhibition. These mechanisms are not equivalent. A decrease in messenger RNA cannot be described as direct enzyme inhibition, and an altered hormone concentration does not identify the affected step without additional evidence.

### 3.2 Bisphenols: direct and indirect effects on specific steroidogenic targets

BPA can interfere with steroidogenesis through both receptor-mediated signaling and direct catalytic effects. In human and rat testicular microsomes, BPA inhibited HSD3B and CYP17A1, while HSD17B3 was affected at higher concentrations. Human enzymes were more sensitive than the corresponding rat enzymes in this system (16). These assays establish biochemical plausibility but used micromolar concentrations and do not demonstrate that direct catalytic inhibition is the dominant mechanism at typical environmental exposure.

Replacement bisphenols show both overlap and divergence. Reporter assays demonstrate differences in estrogen-receptor activation and androgen-receptor antagonism among BPA, BPS, BPF, BPAF, and other analogues (10, 14). In adult human testis explants, individual analogues differentially altered Leydig-cell hormone secretion (15). BPS has been reported to suppress Leydig-cell differentiation and testosterone synthesis through estrogen-receptor and oxidative mechanisms, with changes in CYP11A1, CYP17A1, NR5A1, and HSD17B3-related activity in experimental models (52).

Aromatase provides another example of analogue-specific action. BPA and multiple alternatives inhibited human placental CYP19A1 in microsomal or placental-cell systems, but inhibitory potency varied widely with molecular structure and lipophilicity (17). In granulosa cells, BPA and BPS both disturbed steroid secretion but induced different early transcriptional programs (18). Accordingly, the mechanistic question is not whether replacement bisphenols are universally weaker or stronger than BPA, but which analogue affects which target, at what concentration, in which reproductive tissue, and for how long.

### 3.3 Phthalates: anti-androgenic signaling and effects on steroidogenic enzymes

Phthalate-induced suppression of androgen synthesis involves several levels of the steroidogenic pathway. Human adult testis explants exposed to DEHP or MEHP showed reduced testosterone production, supporting direct human tissue relevance (53). In testicular microsomes, selected phthalates inhibited HSD3B or HSD17B3 with clear structure–activity relationships; many other phthalates showed little or no inhibition, demonstrating that direct catalytic effects cannot be generalized across the class (27).

MEHP can also act upstream of the catalytic enzymes. Fetal testis studies identified CYP17A1 expression and 17,20-lyase activity as sensitive targets, reducing androgen production without uniform suppression of every steroidogenic protein (28). Other experimental studies link MEHP with impaired cAMP-responsive StAR expression and reduced ferredoxin reductase, as well as loss of mitochondrial membrane potential and respiration, thereby limiting CYP11A1-dependent pregnenolone production (29, 30). Thus, lower testosterone may reflect restricted cholesterol delivery, mitochondrial dysfunction, selective enzyme loss, or direct inhibition, depending on the compound and model.

The developmental timing of these effects is critical. Fetal androgen synthesis supports masculinization, testicular descent, and later reproductive capacity. Human epidemiological evidence is strongest for selected phthalate metabolites and androgen-sensitive developmental or semen-related endpoints, but associations are not uniform and repeated exposure measurements are often required (23, 25). Mechanistic precision should therefore complement rather than replace cautious epidemiological interpretation.

### 3.4 MNPs: mainly indirect disruption of steroidogenesis

Convincing direct binding of plastic particles to the catalytic sites of steroidogenic enzymes has not been demonstrated. The available evidence instead supports indirect disruption through cellular uptake, receptor signaling, and organelle stress. In mice and primary Leydig cells, PS microplastics reduced LHR and AC–cAMP–PKA signaling, followed by lower StAR and multiple steroidogenic enzymes and reduced testosterone. LHR overexpression partly rescued these effects, supporting an upstream signaling mechanism (54).

Particle-mediated mitochondrial injury can further restrict steroidogenesis because cholesterol import and CYP11A1 activity depend on intact mitochondrial structure, membrane potential, electron transfer, and ATP supply. Endoplasmic reticulum stress may reduce synthesis and folding of steroidogenic proteins. Experimental ovarian findings also vary by polymer: PS and PET nanoplastics altered follicle growth, oxidative-stress genes, and steroidogenic pathways differently, illustrating why results obtained with one polymer cannot be generalized to all MNPs (55).

The MNP evidence therefore requires disciplined language. Reduced enzyme messenger RNA or protein after particle exposure should be described as downregulation, not direct enzyme inhibition. Demonstration of particle uptake and pathway rescue strengthens causal inference, whereas changes in hormones alone do not identify the initiating molecular event.

## 4 Oxidative stress, inflammation, and reproductive tissue injury

### 4.1 Inflammatory signaling and loss of redox homeostasis

Inflammatory and oxidative responses are closely connected but should not be collapsed into a single generic mechanism. Stress-responsive pathways such as NF-κB can increase interleukin-6, tumor necrosis factor-α, cyclooxygenase-2, and other mediators that alter reproductive microenvironments. Granulosa cells, Sertoli cells, trophoblasts, and hypothalamic cells all integrate immune and endocrine signals, so persistent inflammation can affect hormone production, gamete support, implantation, and placental function (56–58).

Oxidative stress develops when reactive oxygen species generation exceeds antioxidant capacity. Experimental studies of bisphenols and phthalates have reported increased oxidative stress and altered antioxidant defenses (59, 60). For MNPs, redox and mitochondrial responses have been reported in specific particle models and vary with polymer, size, shape, surface properties, and corona characteristics (31–33, 55, 61). Inflammation can suppress steroidogenic gene expression, and oxidative damage can impair STAR function, mitochondrial membranes, and aromatase-dependent hormone synthesis. Conversely, mitochondrial injury and oxidized lipids can further activate inflammatory signaling. This reciprocal amplification explains why redox and inflammatory markers often accompany steroidogenic disruption without proving that every exposure enters the pathway at the same point.

### 4.2 Mitochondrial and endoplasmic reticulum stress, apoptosis, and ferroptosis

Mitochondria are a mechanistic bridge between oxidative injury and endocrine dysfunction. They generate steroid precursors, regulate apoptosis, maintain redox balance, and supply energy for gametogenesis and cellular repair. Oxidative damage can reduce mitochondrial membrane potential, disrupt cristae, alter calcium handling, and decrease ATP production. In steroidogenic cells, these changes restrict cholesterol processing before any direct enzyme inhibition occurs.

When cellular repair is exceeded, regulated cell death converts signaling disturbance into structural tissue loss. Excessive granulosa-cell apoptosis can promote follicular atresia and reduce oocyte support, while germ-cell or Sertoli-cell apoptosis can impair spermatogenesis (62, 63). Ferroptosis is distinct from apoptosis and is driven by iron-dependent accumulation of lipid peroxides; it may also be accompanied by mitochondrial shrinkage and membrane changes. The high polyunsaturated-fatty-acid content of germ-cell and sperm membranes creates particular susceptibility to lipid oxidation (64, 65). Studies using selected MNP models increasingly report oxidative stress, mitochondrial injury, apoptosis, or ferroptosis-related signatures, but the specific pathway should be supported by appropriate molecular and rescue evidence rather than inferred from reactive oxygen species alone.

### 4.3 Reproductive barriers, particle internalization, and transport of associated contaminants

The blood–testis barrier and placental interface maintain specialized reproductive microenvironments. Sertoli-cell tight junctions protect developing germ cells, while placental trophoblast and endothelial barriers regulate maternal–fetal exchange and endocrine signaling (66, 67). MEHP can reversibly disturb blood–testis barrier organization in experimental systems (68). Experimental studies using selected MNP models suggest that particles may interact with epithelial junctions, undergo cellular uptake, or accumulate at barrier surfaces, where local inflammation and oxidative stress may further increase permeability.

A three-dimensional human endometrial model showed barrier alterations and increased collagen deposition after microplastic exposure, providing a human-relevant demonstration of tissue-interface effects while also illustrating the high concentrations often required in current experiments (69). At the placenta, detection studies and experimental models support contact with the maternal–fetal interface, but translocation rates, fetal dose, and functional consequences remain uncertain (37, 70, 71).

Barrier disruption may amplify mixture exposure because a damaged interface permits greater access of soluble chemicals and particles. At the same time, MNP surfaces can adsorb bisphenols, phthalates, metals, and persistent pollutants. This transport effect is plausible, but adsorption in the environment does not guarantee release in a reproductive tissue. Studies should therefore measure both particle-associated and freely available contaminants under biologically relevant conditions.

### 4.4 Epigenetic changes and persistence of effects

Epigenetic effects of EDCs have been extensively reviewed. Their relevance here is narrower: whether exposure-specific changes in DNA methylation, histone regulation, or microRNA expression alter steroidogenic, antioxidant, inflammatory, or developmental genes and persist after exposure. Bisphenols and phthalates have produced such changes in experimental reproductive systems, but affected targets and direction vary by compound, tissue, sex, dose, and developmental window (72–75).

Evidence for MNP-associated epigenetic reprogramming remains predominantly experimental and may be secondary to oxidative or metabolic stress. An epigenetic association does not establish mediation, and effects observed after gestational exposure are not automatically transgenerational because the fetus and developing germ cells may be directly exposed. Human evidence is currently insufficient to claim true transgenerational reproductive inheritance for the exposure classes reviewed here.

## 5 Human reproductive relevance: what is established and what remains inferred

### 5.1 Human evidence for gonadal and gamete-related effects

The human evidence is most mature for phthalates. Systematic evaluations support associations between selected metabolites and male developmental or semen-related endpoints, although results vary by analyte, timing, and study design (23). Human testis explant studies have shown reduced testosterone production following exposure to selected phthalates or metabolites, providing direct ex vivo human-tissue evidence while not establishing population-level causality (53). For replacement bisphenols, human tissue and fertility-clinic studies are fewer. Adult testis explants show analogue-specific endocrine effects, but small sample sizes and experimental exposure conditions limit population inference (15).

MNP evidence has progressed from tissue detection toward exploratory reproductive associations. Particles have been identified in semen or seminal fluid and follicular fluid, with some studies reporting associations with fertilization, oocyte quality, or ovarian reserve (40–42, 44–46). However, small cohorts, methodological heterogeneity, and uncertain exposure timing currently limit causal interpretation.

### 5.2 Ovarian and assisted-reproduction microenvironments

Follicular fluid offers a biologically relevant matrix because it surrounds the oocyte and reflects local steroidogenic and metabolic conditions. Bisphenols and phthalate metabolites have been measured in serum and follicular fluid of women undergoing assisted reproduction (76, 77). Phthalate metabolites in follicular fluid have also been associated with oxidative-stress biomarkers (60). These findings support internal exposure at the ovarian microenvironment, but a single measurement may not represent exposure across folliculogenesis, and ovarian stimulation can alter hormone concentrations and fluid composition.

Recent MNP studies extend this concept but remain methodologically heterogeneous. Associations between PE particles and oocyte quality or fertilization are biologically coherent with experimental findings, although residual confounding and measurement error remain possible (44). MNP detection should therefore be interpreted together with particle characterization and mechanistic evidence rather than as a stand-alone clinical marker.

### 5.3 Placental and perinatal interface

Bisphenols and phthalates reach the maternal–fetal–placental unit. Multiple bisphenol analogues can cross an ex vivo human placental perfusion model, and placental concentrations of phthalate metabolites correlate with maternal urinary exposure to varying degrees (78, 79). These observations provide stronger evidence of fetal-interface exposure than of a defined clinical outcome. Placental endocrine and barrier effects remain compound- and model-specific (80, 81).

MNPs have been detected in placental specimens using spectroscopic and mass-based methods (37, 71), although reported concentrations and polymer distributions vary substantially. Detection supports placental exposure but does not establish fetal translocation or the particle dose responsible for endocrine effects.

### 5.4 Interpretation of causality

Across all three exposure classes, causal interpretation requires alignment of external exposure, internal dose, molecular target, intermediate biomarker, and reproductive outcome. Phthalates have the strongest chain of evidence because human exposure assessment, epidemiology, experimental anti-androgenic mechanisms, and human tissue studies can be considered together. Replacement bisphenols have plausible and sometimes direct mechanisms, but analogue-specific human outcome data are sparse. For MNPs, internal exposure is increasingly documented, while direct human evidence connecting characterized particles to specific endocrine mechanisms and clinical outcomes remains early (**Table 1**).

**Table 1:** Comparative mechanistic evidence for replacement bisphenols, phthalates, and micro-/nanoplastic exposures in human reproductive endocrinology.

Exposure	Human matrices	Steroidogenesis	Stress /inflammation	Barrier effects	Clinical evidence	Evidence summary	References
Replacement bisphenols *(e.g., BPS, BPF, BPAF)*	Urine; blood/serum; placenta; breast milk; follicular fluid	++	+	+	+	Emerging human outcome evidence with moderate mechanistic support. Internal exposure is well documented, but reproductive outcome data remain limited and heterogeneous across analogues, tissues, and exposure windows.	(9, 10, 13–22, 52, 76, 77, 80, 81)
Phthalates	Urine; serum/plasma; follicular fluid; placenta	+++	++	++	+++	Most mature human evidence base. Associations are strongest for male reproductive endpoints, androgen-related developmental markers, hormonal disruption, and semen-related outcomes; female and pregnancy-related findings are also supported but remain more heterogeneous.	(5, 6, 23–30, 53, 60, 68, 76, 79, 80, 89, 90)
Micro-/nanoplastics (MNPs)	Blood; placenta; meconium; breast milk; semen/seminal fluid; testicular tissue; follicular fluid	+	++	++	+	Early and methodologically limited. Human internal exposure is increasingly supported, including in reproductive tissues, but direct clinical reproductive outcome evidence remains limited, heterogeneous, and strongly influenced by particle characterization and analytical methods.	(7, 8, 12, 31–46, 54, 55, 61, 69–71, 82, 92, 93)

+++ well-supported evidence; ++ moderate evidence; + limited or emerging evidence. Ratings are qualitative and reflect the breadth and consistency of human evidence, directness of human reproductive or tissue evidence, mechanistic support, and reproducibility across studies. Clinical evidence ratings prioritize human reproductive outcomes; tissue detection alone is not considered evidence of clinical effect. Direct enzyme inhibition, altered gene or protein expression, pathway-level signaling changes, tissue detection, and clinical associations are treated as distinct forms of evidence. For MNP studies, interpretation should additionally consider polymer identity, particle size and shape, surface properties, ageing, corona formation, additives, associated contaminants, contamination controls, and analytical methods.

The comparative framework therefore separates convergence from equivalence. Shared reductions in testosterone or increases in oxidative markers do not mean that a phthalate metabolite, BPS, and a PE particle act identically. Similarly, absence of mature outcome evidence does not prove safety, but mechanistic plausibility alone should not be presented as established human reproductive harm.

## 6 Research priorities

First, replacement bisphenols require side-by-side evaluation under identical human-relevant conditions. Studies should compare receptor activity, enzyme kinetics, transcriptional effects, metabolism, and tissue exposure across BPA, BPS, BPF, BPAF, and less-studied analogues. Concentration ranges should include measured human levels as well as higher mechanistic doses, with clear separation of effects that occur only at supraphysiological concentrations.

Second, phthalate research should integrate repeated biomonitoring with pathway-specific markers. Urinary metabolites should be linked to steroid profiles, gonadotropins, oxidative biomarkers, and validated reproductive endpoints using designs that address short half-lives and co-exposure. Mixture methods should identify whether several anti-androgenic compounds jointly disrupt the same functional node (11).

Third, MNP studies require methodological standardization. At minimum, investigators should report polymer identity, particle number and mass, size distribution, shape, surface chemistry, ageing, aggregation, corona conditions, additives, endotoxin status, and particle-free controls. Biological-tissue studies require procedural blanks, non-plastic sampling materials where feasible, recovery experiments, and orthogonal polymer confirmation (82). Studies using pristine PS spheres should be described as model-particle experiments rather than generalized evidence for all MNPs.

Fourth, human-relevant platforms are needed to test causality. Ovarian, testicular, endometrial, and placental organoids, ex vivo tissues, and microphysiological barrier systems can connect realistic internal doses to steroidogenic and inflammatory outcomes (83–85). Rescue experiments, targeted enzyme assays, and single-cell approaches should determine whether observed hormone changes result from direct catalytic effects, altered signaling, or loss of steroidogenic cells.

Finally, future studies should examine the interaction between particles and associated contaminants rather than testing each component in isolation. Factorial designs comparing washed particles, particle-free leachates, and combined exposures can distinguish physical, chemical, and transport-related effects. This distinction is essential for identifying effective prevention strategies.

## 7 Clinical, regulatory, and mitigation implications

Current evidence does not support diagnosing an individual patient with infertility caused by a specific plastic-associated exposure. Nevertheless, a focused environmental history may help contextualize unexplained or multifactorial reproductive disorders. A practical history can address major exposure domains, including occupation, repeated medical-device exposure where relevant, food-storage and heating practices, personal-care products, and substantial household or occupational plastic dust or combustion. This information should be used to identify potentially avoidable exposures rather than to assign causation to an individual patient. Counseling should emphasize uncertainty and avoid implying personal blame (86–88).

Chemical substitution and particle mitigation require different language. Substitution is relevant to BPA and selected plasticizers, but alternatives should undergo class-informed hazard assessment to avoid regrettable substitution. MNPs are not replacement chemicals. They arise from production, product shedding, abrasion, ageing, and fragmentation; their control depends on source reduction, reduced emissions, improved material and product design, filtration, waste and wastewater management, and occupational controls.

Current evidence-based counseling options remain limited. Dietary interventions have lowered BPA and DEHP exposure when contact with food packaging was reduced, and broader lifestyle interventions can reduce selected phenol and phthalate biomarkers (89, 90). Practical advice may therefore include avoiding unnecessary heating of food in plastic and reducing avoidable food-contact exposure where feasible, without promising a fertility benefit. By contrast, routine clinical testing for MNP burden, attribution of infertility to a specific plastic-associated exposure, or modification of fertility treatment based on MNP measurements is not currently supported by evidence.

Population-level action is more important than reliance on individual behavior. Regulatory strategies should evaluate chemical classes and realistic mixtures, improve disclosure and assessment of additives, limit avoidable particle release, and support harmonized biomonitoring. Clinical societies can contribute by distinguishing established evidence from emerging concern and by advocating for research standards that permit meaningful risk assessment (91).

## 8 Conclusions

Replacement bisphenols, phthalates, and MNPs are plastic-associated exposures with distinct mechanisms. Bisphenol substitutes may produce BPA-like but analogue-specific endocrine effects; selected phthalates have been shown in experimental and ex vivo models to disrupt androgen synthesis through enzyme, gene-expression, and mitochondrial pathways; MNP-related effects reported in experimental studies are mainly indirect and depend on particle characteristics and associated chemicals. Despite convergence on steroidogenesis, oxidative stress, and inflammatory injury, similar outcomes do not imply equivalent toxicity. Human evidence is strongest for phthalates, emerging for replacement bisphenols, and limited for MNP-related clinical outcomes. Future studies should prioritize standardized particle characterization, human-relevant models, and designs that separate particle effects from leachates and associated contaminants. Clinical guidance should remain cautious, while policy should support safer chemical substitution and MNP source reduction.
